# CLL-Derived Extracellular Vesicles Impair T-Cell Activation and Foster T-Cell Exhaustion via Multiple Immunological Checkpoints

**DOI:** 10.3390/cells11142176

**Published:** 2022-07-12

**Authors:** Martin Böttcher, Romy Böttcher-Loschinski, Sascha Kahlfuss, Michael Aigner, Andreas Gießl, Andreas Mackensen, Ursula Schlötzer-Schrehardt, Thomas Tüting, Heiko Bruns, Dimitrios Mougiakakos

**Affiliations:** 1 Department of Hematology and Oncology, University Hospital Magdeburg, Otto-von-Guericke University Magdeburg, 39120 Magdeburg, Germany; romy.boettcher-loschinski@med.ovgu.de; 2 Institute of Molecular and Clinical Immunology, Medical Faculty, Otto-von-Guericke University Magdeburg, 39120 Magdeburg, Germany; sascha.kahlfuss@med.ovgu.de; 3 Health Campus Immunology, Infectiology, and Inflammation (GCI3), Medical Center, Otto-von-Guericke University Magdeburg, 39120 Magdeburg, Germany; thomas.tueting@med.ovgu.de; 4 Institute of Medical Microbiology and Hospital Hygiene, Medical Faculty, Otto-von-Guericke University Magdeburg, 39120 Magdeburg, Germany; 5 ChaMP, Center for Health and Medical Prevention, Otto-von-Guericke University Magdeburg, 39120 Magdeburg, Germany; 6 Medical Department 5–Hematology and Oncology, University Hospital Erlangen, Friedrich-Alexander-University of Erlangen-Nürnberg, 91054 Erlangen, Germany; michael.aigner@uk-erlangen.de (M.A.); andreas.mackensen@uk-erlangen.de (A.M.); heiko.bruns@uk-erlangen.de (H.B.); 7 Department of Ophthalmology, University Hospital Erlangen, Friedrich-Alexander-University of Erlangen-Nürnberg, 91054 Erlangen, Germany; andreas.giessl@uk-erlangen.de (A.G.); ursula.schloetzer-schrehardt@uk-erlangen.de (U.S.-S.); 8 Laboratory for Experimental Dermatology, Department of Dermatology, Medical Faculty, Otto-von-Guericke University Magdeburg, 39120 Magdeburg, Germany

**Keywords:** chronic lymphocytic leukemia, extracellular vesicles, T-cells, immune evasion, immune checkpoints, cellular therapy

## Abstract

*Background:* Chronic lymphocytic leukemia (CLL) is characterized by the clonal expansion of malignant B-cells and multiple immune defects. This leads, among others, to severe infectious complications and inefficient immune surveillance. T-cell deficiencies in CLL include enhanced immune(-metabolic) exhaustion, impaired activation and cytokine production, and immunological synapse malformation. Several studies have meanwhile reported CLL-cell–T-cell interactions that culminate in T-cell dysfunction. However, the complex entirety of their interplay is incompletely understood. Here, we focused on the impact of CLL cell-derived vesicles (EVs), which are known to exert immunoregulatory effects, on T-cell function. *Methods:* We characterized EVs secreted by CLL-cells and determined their influence on T-cells in terms of survival, activation, (metabolic) fitness, and function. *Results:* We found that CLL-EVs hamper T-cell viability, proliferation, activation, and metabolism while fostering their exhaustion and formation of regulatory T-cell subsets. A detailed analysis of the CLL-EV cargo revealed an abundance of immunological checkpoints (ICs) that could explain the detected T-cell dysregulations. *Conclusions:* The identification of a variety of ICs loaded on CLL-EVs may account for T-cell defects in CLL patients and could represent a barrier for immunotherapies such as IC blockade or adoptive T-cell transfer. Our findings could pave way for improving antitumor immunity by simultaneously targeting EV formation or multiple ICs.

## 1. Introduction

Chronic lymphocytic leukemia (CLL) is the leukemia with the highest incidence amongst adults in Western civilizations [1]. It is a disease of the elderly with a median age of 70 at diagnosis. It is characterized by a clonal expansion of antigen-experienced, malignant CD5^+^ B-cells that accumulate in the peripheral blood, bone marrow, and lymph nodes [2] where they undergo recurrent bi-directional interactions with other components of the immune system, especially T-cells [3]. This leads, amongst others, to severe immune defects resulting in infectious complications and an inefficient mounting of an anti-tumor immune response. Despite tremendous research efforts in past decades, CLL remains incurable, since the only curative option is allogeneic hematopoietic stem cell transplantation, which is not applicable to most patients due to their advanced age and its toxicity [4]. Recent advances include BTK- (such as ibrutinib), Bcl2- (such as Venetoclax), and PI3K-inhibitors (such as idelalisib), as well as cellular therapies such as CD19-directed CAR-T-cells [5]. Although these novel therapeutics show promising results, there are still obstacles to overcome. First and foremost, a better understanding of the interplay between CLL-cells and their microenvironment could help improving therapeutic efficacy.

The T-cell compartment in CLL is altered on two levels, which is beneficial for the malignant cells: T-cells act in a pro-tumorigenic manner and display an impaired anti-CLL cell activity. On the one hand, CLL T-cells in secondary lymphoid organs promote survival and proliferation of CLL-cells in so-called “pseudofollicles” by providing CD40/CD40L interaction, and secreting cytokines such as IL-4, IFNγ, and IL-21 [6]. On the other hand, although numerically increased and skewed towards the CD8^+^ subset, circulating CLL T-cells are characterized by functional defects including immune synapse malformation, alterations in cytokine secretion, and a general cytotoxic insufficiency [7]. Underlying mechanisms of this complex situation are still insufficiently understood. In particular, cell-contact-independent intercellular communication remains incompletely explored. However, a better understanding of the factors causing T-cell dysfunctions could help in two ways: interference with pro-CLL effects and restoration of immune competence.

Extracellular vesicles (EVs) are membranous nanoparticles that can carry different cargoes, including DNA, RNA, and membrane and soluble proteins, and serve, amongst others, as intercellular communicators [8,9]. They are characterized by a size of 30–200 nm and the tetraspanins CD9, CD63, and CD81 as prototypical markers [10]. A pathogenic role of EVs has been described in several disorders including infections, autoimmunity, and cancer. In CLL, EV secretion was found to be regulated by B-cell-receptor signaling [11,12]. To date, research studying EVs in CLL has mainly been focused on their use as biomarkers [13], and characteristic signatures of mRNA and microRNA carried by EVs were found to be of potential prognostic relevance [11,14]. Furthermore, CLL-EVs were studied in terms of intercellular communication between the malignant B-cells and their microenvironment including monocytes [15] and stroma cells [16]. However, only a few studies examining the effects of CLL-derived EVs on T-cells are available including a manuscript presenting evidence of the inhibitory properties of CLL-EVs on CD19-directed CAR-T-cell therapy [17], which will be discussed here as well.

We hypothesized that CLL-EVs may add to the mechanisms of T-cell dysfunctions by intercellular transfer of bioactive factors. Thus, we aimed to comprehensively study the effects of CLL-EVs on T-cell biology. To this end, we isolated EVs from CLL cell lines and CLL patient plasma that we characterized and utilized in healthy donor (HD)-derived T-cell cultures. We found that CLL-EVs are capable of interfering with T-cell survival and proliferation, inhibiting T-cell activation and cytokine secretion, dampening the overall metabolic activity, fostering T-cell exhaustion, inducing regulatory T-cells (TRegs), and blocking the formation of immunological synapses. We identified a plethora of immune checkpoints (ICs) carried by the CLL-EVs (including the ectonucleotidases CD39 and CD73, PD-L1, TGFβ, the TIM3 ligand galectin-9, and the TIGIT ligand CD112) that could explain the observed effects on T-cells. Ultimately, we confirm and append previous findings about the detrimental influence of CLL-EVs on CAR-T-cell function [17]. Our novel findings on CLL-EVs could contribute to developing the means for restoring T-cell immunity in CLL.

## 2. Materials and Methods

### 2.1. Cell Lines and Cells

Peripheral blood and plasma samples were collected upon approval by the local ethics committee (approval numbers: 291_14B, 289_16B) and patients’ written informed consent. Human CLL cell lines MEC-1, HG-3, EHEB, and PGA1, and the cell lines Raji and Jurkat were purchased from DSMZ (Braunschweig, Germany). EBV-LCL lines were kindly provided by Dr. Regina Gary and Dr. Sascha Kretschmann (Erlangen, Germany).

### 2.2. Extracellular Vesicles

Isolation: CLL cell culture supernatants were harvested, sequentially centrifuged, and filtered (0.22 µm mesh) to remove large vesicles, apoptotic bodies, and debris. Subsequently, EVs were isolated by ultracentrifugation (70 min, 110,000× *g*, 4 °C), washed once with PBS, and stored in PBS at −80 °C until further use. EVs from treatment-naïve CLL patient plasma (*n* = 8) were similarly isolated by sequential centrifugation and filtration (0.22 µm mesh) for removal of large vesicles, apoptotic bodies, and debris, and subsequently harvested by ultracentrifugation at 110.000× *g* once for 2 h and twice for 70 min. For quantification, total protein content was used and analyzed by a Pierce BCA Assay kit (ThermoFisher Scientific, Waltham, MA, USA).

Electron microscopy: Fixed EV solutions (4% PFA in PBS mixed 1:1 with EV) were placed onto 10 min UV irradiated 300-mesh formvar/carbon-coated grids and allowed to absorb to the formvar for 5 min. Grids were incubated 5 min in a dry environment, rinsed one time with PBS and placed in 1% glutaraldehyde (in PBS) for 5 min. After rinsing in distilled water (7 times), the grids were stained for contrast using a 2% uranyl oxalate solution (pH 7 for 5 min in dark). Afterwards the grids were incubated in drops of methyl cellulose–uranyl acetate (8 parts 2% methyl cellulose (in water), 1 part ddH_2_O, 1 part 4% uranyl acetate (in water), pH 4, sterile filtered) for 10 min on ice (dark) [18]. After that, grids were removed with stainless steel loops and excess fluid was blotted by gentle pushing on Whatman filter paper. After air-drying, the samples were examined and photographed with a Zeiss EM10 electron microscope (Zeiss, Jena, Germany) and a Gatan SC1000 Orius™ CCD camera (GATAN, Munich, Germany) in combination with the DigitalMicrograph™ software 3.1 (GATAN, Pleasanton, CA, USA). Images were adjusted for contrast and brightness using Adobe Photoshop CC 2018 (Adobe Systems, San José, CA, USA).

Fluorescent labeling: Cells or EVs were labeled for uptake experiments using the PKH26 Red Fluorescent Cell Linker Mini kit (Sigma-Aldrich, St. Louis, MO, USA) according to the manufacturer’s instructions. Excess dye was quenched by adding 2 mL 10% BSA in PBS and a subsequent washing step with PBS by ultracentrifugation (70 min, 100,000× *g*, 4 °C).

### 2.3. Cell Culture

Human CLL cell lines MEC-1, HG-3, PGA1, and EHEB were grown as high-density cell culture in CELLine classic bioreactor flasks (Sigma-Aldrich) in RPMI-1640 (Sigma-Aldrich) supplemented with 2 mM L-glutamine (Sigma-Aldrich), 10% exosome-depleted FCS (c.c.pro, Vogtei, Germany), and 40 U/mL penicillin–streptomycin (ThermoFisher Scientific) at 37 °C, 5% CO_2_. Jurkat and Raji cells were cultured in AIM V (ThermoFisher Scientific) in conventional cell culture flasks at 37 °C, 5% CO_2_. Cell line cultures were split twice per week.

Healthy donor peripheral blood mononuclear cells (PBMCs) were obtained from a total of 50 volunteers using Ficoll-Paque (GE Healthcare, Chalfont St. Giles, UK). T-cells were isolated from human PBMCs using the Pan T cell isolation kit, human (Miltenyi Biotec, Bergisch-Gladbach, Germany) according to manufacturer’s guidelines. T-cells or CAR-T-cells were seeded at a density of 10^6^/mL in AIM V or AIM V with 50 U/mL IL-2 (Miltenyi Biotec), respectively, and pretreated with 30 µg/mL CLL-EVs for 3 days. Afterwards, cells were re-seeded at the same density with fresh EVs in the presence or absence of anti-CD2/CD3/CD28-coated beads (Miltenyi Biotec) for 24–96 h depending on the subsequent analysis.

Raji and Jurkat cells were co-cultured at a ratio of 1:4 in AIM V. Raji cells were pre-incubated with 30 ng/mL SEA for 45 min. The co-culture was incubated for 24 h in the absence/presence of 30 µg/mL CLL-EVs. For selected experiments EVs at a density of 800 µg/mL were pre-incubated with 10 µg/mL anti-PDL1 antibody (ThermoFisher Scientific) or the corresponding isotype control for 30 min. Secreted IL-2 was quantified with a pre-made ELISA kit (Biolegend, San Diego, CA, USA) according to the manufacturer’s instructions.

The inhibitors used in all experiment are listed in Supplementary Table S1.

### 2.4. Flow Cytometry

Samples were recorded on a FACS Canto II (BD Biosciences, Franklin Lakes, NJ, USA) and data was analyzed using FlowJo V 10 (BD Biosciences).

Multiplex assay: Common EV markers were analyzed by flow cytometry using a commercial MACSPlex exosome kit (Miltenyi Biotec) according to the manufacturer’s instructions.

EV flow cytometry: To analyze membrane and soluble proteins carried by EVs, the EVs or their lysates were coated on 4 µm latex beads (ThermoFisher Scientific) at 4 °C overnight, free binding sites were saturated by 1 M glycine (Sigma-Aldrich), and beads were washed with PBS/5% BSA. Then, CLL-EV-coated beads were incubated with human IgG (Gamunex, Grifols, Barcelona, Spain) and stained using fluorochrome-conjugated antibodies and the respective isotype controls (see Supplementary Table S2).

PKH26 EV-uptake assay: PKH26-labeled CLL-cells were incubated with activated and non-activated healthy donor T-cells in the presence and absence of EV-secretion inhibitors (Nexinhib20, GW4869) at indicated ratios for 24 h.

PKH26-labeled EVs were incubated with activated and non-activated healthy donor T-cells in the presence and absence of EV uptake inhibitors (EIPA, cytochalasin D, dynasore, and heparin). Additionally, after incubation with labeled EVs, T-cells were treated with trypsin prior to analysis to distinguish between surface-bound and internalized EVs.

Proliferation: For proliferation analysis, cells were stained with violet proliferation dye 450 (BD Biosciences) prior to the culture according to the manufacturer’s recommendation. Dye dilution was recorded and analyzed using the proliferation tool in FlowJo.

Surface staining: Samples were stained with Zombie Aqua (Biolegend) for dead cell discrimination, blocked with human IgG (Gamunex), and stained with fluorochrome-conjugated antibodies (see Supplementary Table S2) against surface proteins according to the manufacturer’s instruction.

Intracellular staining: Intracellular proteins were stained subsequent to surface staining using the FoxP3 transcription factor staining Kit (Biolegend) and fluorochrome-conjugated antibodies (see Supplementary Table S2) according to the manufacturer’s manual.

PhosFlow: Intracellular phospho-proteins were stained using the PhosFlow^TM^ Fix buffer I together with the PhosFlow^TM^ Perm buffer III (both BD Biosciences) and fluorochrome-conjugated phospho-specific antibodies (see Supplementary Table S2) according to the manufacturer’s recommendations.

Glucose uptake: Glucose uptake was analyzed by the use of 6-NBDG (6-(N-(7-nitrobenz-2-oxa-1,3-diazol-4-yl)amino)-6-deoxyglucose; ThermoFisher Scientific). Cells were washed in PBS and glucose-free medium by centrifugation (300× *g*, 5 min, 4 °C) and resuspended in glucose-free medium containing 0.3 mM 6-NBDG. Samples were incubated for 15 min at 37 °C, 5% CO_2_ and subsequently recorded after two washing steps.

Fatty acid uptake: Uptake of long-chain fatty acids was determined by staining with Bodipy^TM^ FL C_16_ (ThermoFisher Scientific) according to the product’s manual.

Mitochondrial parameters: Mitochondrial biomass and membrane potential were determined with MitoTracker^TM^ Green and tetramethylrhodamine ethyl ester (TMRE) (both ThermoFisher Scientific) according to the manufacturer’s manual.

Reactive oxygen species: Cellular reactive oxygen species and mitochondrial superoxides were analyzed using CellROX Deep Red reagent and MitoSOX Red (both ThermoFisher Scientific) according to the manufacturer’s manual.

### 2.5. Fluorescent Microscopy

Isolated T-cells were activated with plate-bound CD3/soluble CD28 (both ThermoFisher Scientific) in the presence of PKH26-labeled CLL-EVs for 48 h at 37 °C, 5% CO_2_. Cells were harvested and coated onto poly-D-lysine microscopic slides (ThermoFisher Scientific). Slides were washed with PBS and cells were fixed with 4% paraformaldehyde solution. After washing, slides were blocked with ratIgG, incubated with anti-CD4-AF488 or anti-CD8-AF488 antibodies, and counter-stained with DAPI. Samples were recorded on a Leica laser-scanning microscope and images were analyzed using LAS X Core software version 3 (Leica, Wetzlar, Germany).

### 2.6. Metabolic Analyses

Glucose/lactate: Glucose and lactate concentrations in cell culture supernatants were quantified using a SuperGL_compact_ (Hitado, Möhnesee, Germany) according to the device’s manual.

Extracellular flux analysis: Bioenergetics of glycolysis and mitochondrial respiration were analyzed on the extracellular flux analyzer Seahorse XFe 96 (Agilent, Santa Clara, CA, USA), as previously described [19].

Scenith: Scenith analyses and calculations were performed according to the original publication [20].

ATP/Adenosine assay: ATP concentrations in supernatants were quantified with a fluorometric ATP Assay Kit (Sigma-Aldrich). Adenosine concentrations in supernatants were quantified with a fluorometric Adenosine Assay Kit (PromoCell, Heidelberg, Germany).

### 2.7. Imaging Cytometry

Imaging cytometry was performed on a FlowSight (Luminex, Austin, TX, USA) and data were analyzed using IDEAS 6.3 including a machine-learning module (Luminex).

For immune synapse staining HD-derived, isolated T-cells were pre-stimulated with anti-CD2/CD3/CD28-coated beads (Miltenyi Biotec) in the absence/presence of 30 µg/mL CLL-EVs for 48 h at 37 °C, 5% CO_2_. Raji cells were labeled with CFSE (ThermoFisher Scientific) and loaded with 1 µg/mL SEA and SEB (both Sigma-Aldrich). T-cells and Raji cells were mixed at a ratio of 1:1, CLL-EVs were added at 60 µg/mL, and samples were incubated for 7 min at 37 °C. Samples were fixed with 1.5% PFA and permeabilized with PBS/2%FCS/0.1% Triton-X. They were stained with surface antibodies and Biotin-XX-Phalloidin (ThermoFisher Scientific), as well as Streptavidin-PE/TexasRed (ThermoFisher Scientific). Staining of nuclei was done using either 7-AAD (Biolegend) or Syto13 (ThermoFisher Scientific). Synapse staining of CAR-T-cells was similarly performed using Mec-1 cells as the target instead of Raji with a CD19 surface staining for detection.

### 2.8. Statistics

Outliers were determined using the ROUT test. Differences in means were evaluated with parametric (paired/unpaired t-test, one-way ANOVA) or nonparametric (unpaired Mann–Whitney, paired Wilcoxon, unpaired Kruskal–Wallis) tests based on the number of comparisons (two or more than two) and distribution levels (as determined by Shapiro–Wilk and Kolmogorov–Smirnov). All statistical analyses were performed using GraphPad Prism Version 7 or 8 (GraphPad Software Inc., San Diego, CA, USA) at a significance level of *p* < 0.05.

## 3. Results

### 3.1. CLL-Cells Secrete EVs That Interact with T-Cells

**Figure 1:**
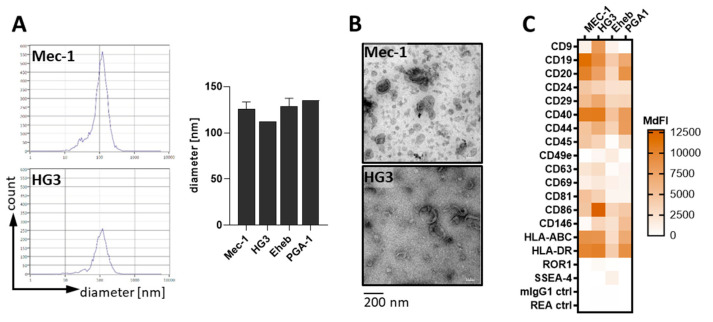

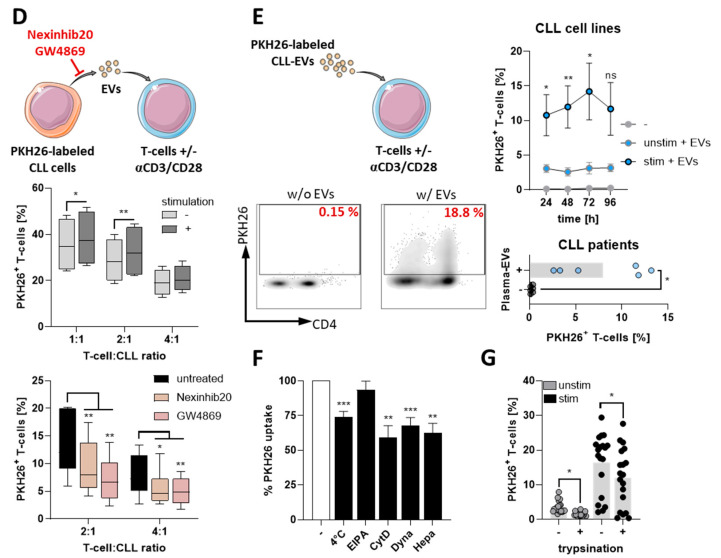
CLL-cells secrete EVs that attach to and enter T-cells. (**A**) Isolated EVs from the CLL cell lines (Mec-1 *n* = 4, HG3 *n* = 1, Eheb *n* = 4, and PGA1 *n* = 1) were analyzed for their diameter by nanoparticle tracking (ZetaView^®^) as representatively shown on the left and quantified on the right. (**B**) CLL-EVs were visualized using electron microscopy. (**C**) Membrane-bound markers on CLL-EVs were analyzed using a flow-cytometry-based multiplex assay (*n* = 3–5). Values are shown as the median fluorescence intensity (MdFI). (**D**) Top panel: healthy donor (HD)-derived T-cells were cultured together with PKH26-labeled CLL cell lines at indicated cell rations for 24 h in the absence(-)/presence(+) of activating anti-CD2/CD3/CD28-coated beads and analyzed for the PKH26 signal by means of flow cytometry (*n* = 4). Bottom panel: anti-CD2/CD3/CD28-stimulated HD-derived T-cells were cultured together with PKH26-labeled CLL cell lines at indicated cell rations for 24 h in the absence/presence of inhibitors for vesicle secretion (Nexinhib20, GW4869) and analyzed for the PKH26 signal by means of flow cytometry (*n* = 6). (**E**) HD-derived T-cells were cultured with isolated, PKH26-labeled CLL-EVs from cell lines (top right, *n* = 4) for indicated times or patient plasma (bottom right, *n* = 6) for 24 h and analyzed for the frequency of PKH26^+^ T-cells by flow cytometry (representative density plot on the left). (**F**) CLL-EV uptake by T-cells was further analyzed after 6 h under different conditions as indicated and depicted in relation to untreated (-, set as 100%, *n* = 11). (**G**) HD-derived T-cells that were incubated with PKH26-labeled CLL-EVs for 24 h were trypsinized before the analysis to erase surface-bound EVs and compared to untrypsinized controls (*n* = 19). Error bars show the standard error mean. * *p* < 0.05, ** *p* < 0.01, *** *p* < 0.001, ns not significant.

T-cells are central to tumor immune surveillance. Thus, we wanted to further explore the contact-independent cross-talk between CLL-cells and T-cells, with a focus on EVs. First, we isolated EVs from different CLL cell lines (Mec-1, HG3, PGA1, and Eheb) as well as CLL-patient plasma by sequential (ultra-)centrifugation. We confirmed typical characteristics of the EVs, such as a diameter of 100–150 nm, shape, and EV-specific protein markers, using a nanoparticle tracking analyzer (ZetaView^®^, Figure 1A), electron microscopy (Figure 1B), and a flow cytometry-based multiplex assay (Figure 1C), respectively. The latter showed, amongst others, the presence of typical EV-markers such as CD9, CD63, and CD81, as well as markers of the parental cell type such as CD19, CD20, and CD40, some of which we could confirm on EVs obtained from the CLL-patient plasma (Supplementary Figure S1A).

To investigate a potential EV-mediated communication between CLL-cells and T-cells, we co-cultured PKH26-labeled CLL cell lines (that also secrete PKH26^+^ EVs, data not shown) together with HD-derived T-cells in the absence/presence of a T-cell stimulatory signal (via anti-CD2/CD3/CD28-coated beads). We found that T-cells obtained a PKH26 signal after 24 h of co-culture, which was significantly stronger upon T-cell stimulation. However, the PKH26 signal was significantly reduced by inhibitors of EV secretion used during the co-culture (i.e., Nexinhib20 and GW4869) indicating a transfer of EVs from CLL-cells to T-cells (Figure 1D). Isolated cell-line- and patient-plasma-derived PKH26-labeled CLL-EVs were efficiently taken up by or bound to T-cells, which was most prominent upon T-cell stimulation (Figure 1E). Notably, CD4^+^ and CD8^+^ T-cells were similarly capable of interacting with CLL-EVs (Supplementary Figure S1B). The analysis of CLL-EV uptake by T-cells under different conditions including low temperature and several inhibitors of uptake routes [21] revealed that interaction of T-cells with CLL-EVs was only partly energy-dependent and mainly occurred via endocytosis (Figure 1F). Moreover, labeled CLL-EVs are not only found inside, but also as mentioned previously, on the outer cell membrane of T-cells (Supplementary Figure S1C). To estimate the amount of surface-bound CLL-EVs, we trypsinized the T-cells after culture in presence of CLL-EVs to detach surface-bound EVs and detected a significant reduction of the PKH26 signal (Figure 1G).

Together, these data show that CLL-cells secrete EVs that potentially bind to the T-cell surface and/or enter T-cells.

### 3.2. CLL-EVs Impair T-Cell Survival, Proliferation, and Activation

**Figure 2:**
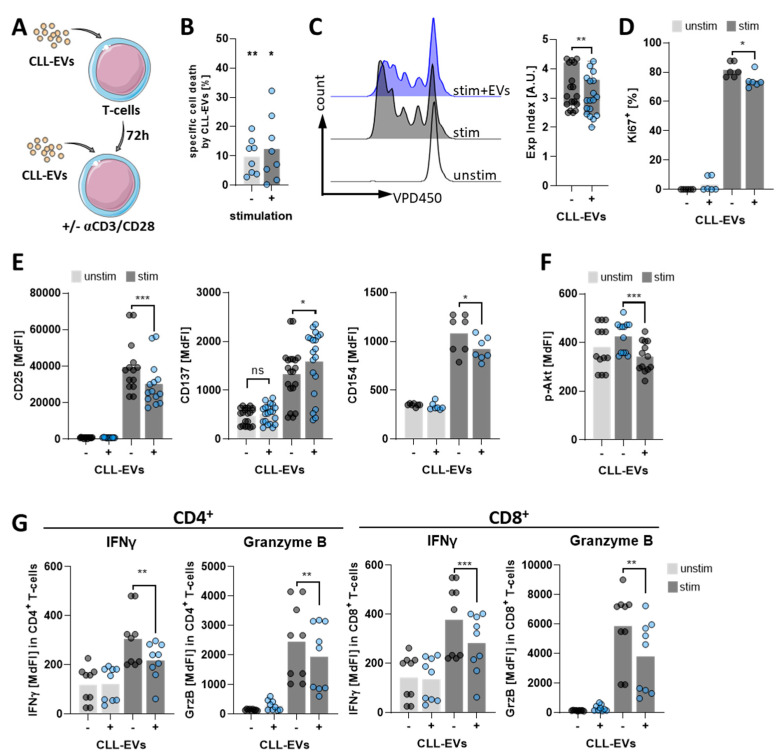
CLL-EVs affect T-cell survival, proliferation, and activation. (**A**) Scheme showing the experimental setup: T-cells were pre-cultured for 72 h in the absence/presence of CLL-EVs and subsequently supplemented with fresh CLL-EVs and cultured with/without stimulating anti-CD2/CD3/CD28-coated beads for additional 24–96 h, as described below. (**B**) Viability of CLL-EV-treated T-cells was assessed by flow cytometry and compared to untreated ones after 72 h of culture. Specific cell death by the EV-treatment was calculated and is depicted as the percentage (*n* = 8). (**C**) To analyze proliferation in the absence/presence of CLL-EVs, T-cells were labeled with the violet proliferation dye 450 (VPD450) and dye dilution was measured after 96 h of culture, as representatively shown on the left. The quantification on the right depicts the expansion index as calculated using the proliferation tool in FlowJo (*n* = 18). (**D**) To assess cell division the frequency of Ki67^+^ T-cells was analyzed by intracellular flow cytometry after 72 h of culture in the absence/presence of CLL-EVs (*n* = 6). (**E**) Early activation markers CD25 (*n* = 14), CD137 (*n* = 20), and CD154 (*n* = 7) were analyzed by flow cytometry on T-cells after 24 h of culture in the absence/presence of CLL-EVs. (**F**) TCR downstream signaling was analyzed by PhosFlow of Akt in T-cells cultured for 24 h in the absence/presence of CLL-EVs (*n* = 13). (**G**) Expression levels of interferon gamma (IFNγ) and granzyme B (GrzB) were determined by intracellular flow cytometry in T-cells cultured for 72 h in the absence/presence of CLL-EVs (*n* = 9). * *p* < 0.05, ** *p* < 0.01, *** *p* < 0.001, ns: not significant. MdFI: median fluorescence intensity.

Next, we aimed to analyze the consequences of the interaction between CLL-EVs and T-cells. Therefore, we cultured T-cells for 3 days in the absence/presence of CLL-EVs and subsequently stimulated them with anti-CD2/CD3/CD28-coated beads together with a second addition of fresh CLL-EVs (Figure 2A). We detected an EV-triggered increase of specific cell death (Figure 2B) as well as a reduction in proliferation (Figure 2C) and cell division (Figure 2D). Moreover, T-cell activation, as exemplified by upregulation of activation markers (Figure 2E) and downstream TCR signaling (i.e., AKT pathway) (Figure 2F), was significantly attenuated by CLL-EVs. We found that the typical early activation markers CD25 and CD154 were both significantly less abundant in presence of CLL-EVs. However, not all activation markers seemed to be uniformly affected, as CD137 was slightly but significantly induced by CLL-EVs. Nevertheless, production of the key effector cytokine IFNγ and the cytolytic granzyme B was also significantly hampered by CLL-EVs both in CD4^+^ and CD8^+^ T-cells (Figure 2G).

### 3.3. CLL-EVs Alter T-Cell Metabolism and Skew Their Subset Composition

**Figure 3:**
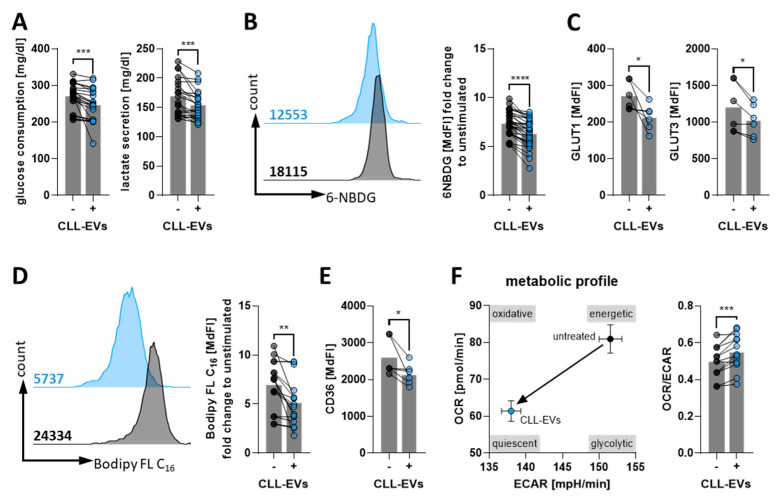

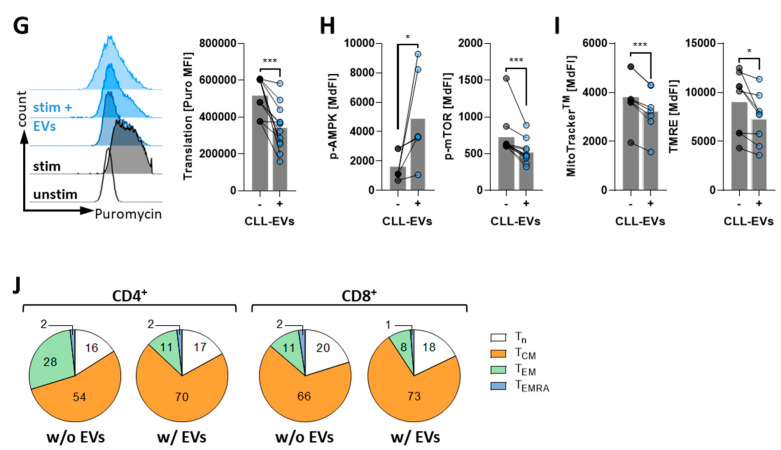
CLL-EVs modulate T-cell metabolism. (**A**) Consumption of glucose and secretion of lactate in the supernatant of activated T-cells cultured for 72 h in the absence/presence of CLL-EVs was quantified (*n* = 21). (**B**) Glucose uptake of activated T-cells cultured for 72 h in the absence/presence of CLL-EVs was semi-quantified by flow cytometry using the fluorescent glucose analogue 6-NBDG and depicted as the median fluorescence intensity as representatively shown on the left and summarized on the right (*n* = 30). (**C**) Surface levels of glucose transporters 1 and 3 were analyzed by flow cytometry on activated T-cell culture for 72 h in the absence/presence of CLL-EVs (*n* = 6). (**D**) Long-chain fatty acid (LC-FA) uptake of activated T-cells cultured for 72 h in the absence/presence of CLL-EVs was semi-quantified by flow cytometry using the fluorescent LC-FA analogue Bodipy^®^ FL C_16_ and depicted as the median fluorescence intensity as representatively shown on the left and summarized on the right (*n* = 16). (**E**) Surface levels of fatty acid transporter CD36 was analyzed by flow cytometry on activated T-cell culture for 72 h in the absence/presence of CLL-EVs (*n* = 6). (**F**) The metabolic profile of activated T-cell culture for 72 h in the absence/presence of CLL-EVs was recorded by metabolic flux analysis. Extracellular acidification rate (ECAR, as a surrogate for aerobic glycolysis) and oxygen consumption rate (OCR, as a surrogate for oxphos) were determined in real-time and plotted as depicted in the left graph. The balance between oxphos and glycolysis is shown as the ratio of OCR/ECAR in the right graph (*n* = 9). (**G**) The level of translation as an indicator of metabolic activity/energy production was measured by flow cytometry in activated T-cells cultured for 72 h in the absence/presence of CLL-EVs (*n* = 12). (**H**) AMPK (*n* = 6) and mTOR (*n* = 11) signaling were analyzed by PhosFlow in activated T-cells cultured for 24 h in the absence/presence of CLL-EVs. (**I**) Mitochondrial biomass (MitoTracker^TM^, *n* = 7) and mitochondrial membrane potential (TMRE, *n* = 8) were measured in activated T-cells after 72 h of culture in the absence/presence of CLL-EVs by flow cytometry. (**J**) T-cell-subset frequencies of naïve (T_n_), central memory (T_CM_), effector memory (T_EM_), and terminally differentiated (T_EMRA_) ones were analyzed in activated CD4^+^ and CD8^+^ T-cells by flow cytometry after 72 h of culture in the absence/presence of CLL-EVs (*n* = 5–6). Error bars show the standard error mean. * *p* < 0.05, ** *p* < 0.01, *** *p* < 0.001, **** *p* < 0.0001. MdFI: median fluorescence intensity.

The prominent role of metabolism in governing the growth, activation, differentiation, and function of T-cells has become evident during recent years [22,23]. Thus, we aimed to identify potential CLL-EV-induced changes of the metabolic T-cell phenotype. Analyzing the T-cells’ supernatant, we noticed that both glucose consumption and secretion of lactate, as surrogates for aerobic glycolysis, are significantly reduced when T-cells are stimulated in presence of CLL-EVs (Figure 3A). Accordingly, glucose uptake, as analyzed by the use of a fluorescent glucose analogue (6-NBDG), was also significantly reduced (Figure 3B). In line with reduced glucose uptake (and its utilization), expression of glucose transporter 1 and 3 (GLUT1/3) was significantly lower in T-cells treated with CLL-EVs (Figure 3C). At the same time, fatty acid metabolism seemed to be affected as well, since uptake of long-chain fatty acids (FAs) (as measured by the fluorescent FA analogue Bodipy FL C_16_, Figure 3D) and the respective FA transporter CD36 (Figure 3E) were both significantly reduced in response to treatment with CLL-EVs. In fact, the overall metabolic profile of the CLL-EV-treated T-cells was shifted towards a more quiescent state with a concordant reduction of glycolytic and oxidative phosphorylation (oxphos) activity as assessed by metabolic flux analyses (Figure 3F, Supplementary Figure S2A). However, the overall equilibrium between oxphos and glycolysis was slightly but significantly shifted towards oxphos (Figure 3F). As recently described, the level of protein translation directly correlates with the cellular metabolic activity [20]. In accordance to our observations from the metabolic flux analyses, protein translation level, which can be measured based on puromycin incorporation in elongating peptides, in activated T-cells was significantly diminished in presence of CLL-EVs (Figure 3G). Furthermore, we noticed that dependence on oxphos for meeting the T-cells’ bioenergetic demands was lower upon CLL-EV treatment (Supplementary Figure S2B). Activity of key metabolic regulators [24] analyzed by PhosFlow was shifted towards a non-proliferative, resting state as shown by increased AMPK and decreased mTOR phosphorylation (including its downstream signaling components p-S6 and p-4EBP1) (Figure 3H, Supplementary Figure S2C). As mTOR signaling promotes mitochondrial biogenesis while AMPK signaling maintains mitochondrial homeostasis, we detected a reduced mitochondrial biomass together with a lower mitochondrial membrane potential (Figure 3I). Total cellular and mitochondrial reactive oxygen species (ROS) levels were also found reduced. Both effects (reduced mitochondrial membrane potential and reduced ROS levels) can reflect the overall reduced metabolic activity or mitochondrial mass or (more likely) a combination of both CLL-EV-triggered phenomena (Supplementary Figure S2D).

It is well-established that T-cell subsets display different metabolic phenotypes [25]. Thus, we determined the frequencies of naïve (n; CD45RO^−^ CCR7^+^), central memory (CM; CD45RO^+^ CCR7^+^), effector memory (EM; CD45RO^+^ CCR7^−^), and terminally differentiated effector T-cells (EMRA; CD45RO^−^ CCR7^−^) [26] (gating strategy see Supplementary Figure S2E) in absence or presence of CLL-EVs. It is known that central memory T-cells are less proliferative and metabolically active with a preference for oxphos while effector memory T-cells rapidly proliferate and display an active glycolytic metabolism [25]. Thus, it is not surprising that we found a skewing of effector/memory distribution towards central memory cells at the expanse of effector memory cells by the presence of CLL-EVs (Figure 3J).

Taken together, these data reveal a substantial modulation of the T-cell bioenergetics by CLL-EVs.

### 3.4. CLL-EVs Promote T-Cell Exhaustion and Regulatory T-Cell Formation

Bioenergetic alterations have been shown to not only directly act in a T-cell suppressive manner but to also favor a tolerogenic milieu by promoting T-cell exhaustion [27] and/or Treg induction [28].

**Figure 4:**
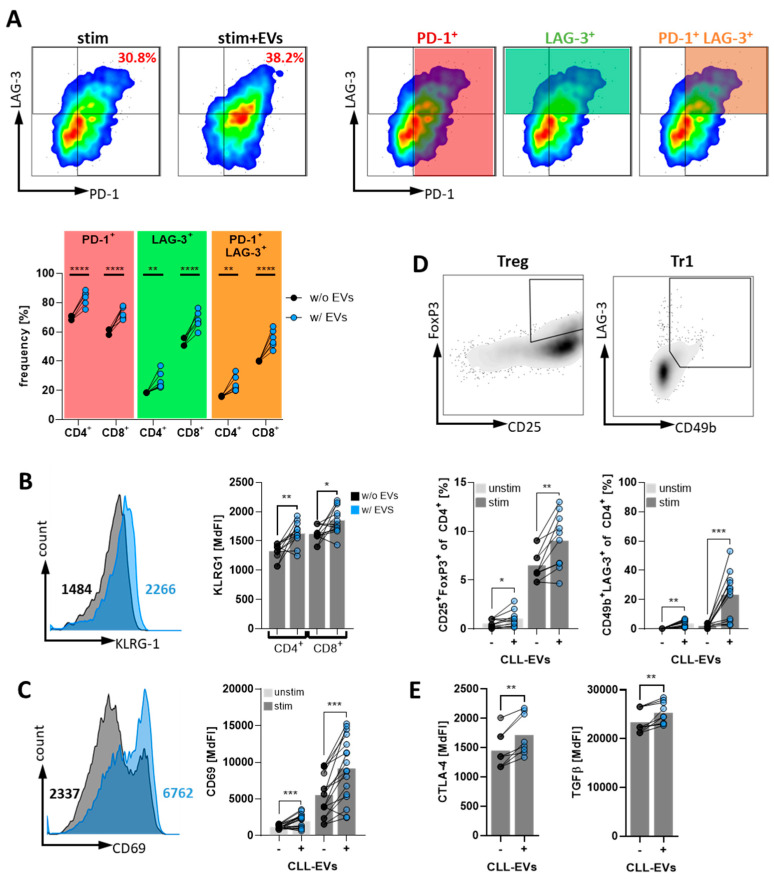
CLL-EVs promote T-cell exhaustion and regulatory T-cell formation. (**A**–**C**) The exhaustion markers PD-1 and LAG-3 (**A**, *n* = 6), KLRG1 (**B**, *n* = 12), and CD69 (**C**, *n* = 17) were analyzed on the surface of activated CD4^+^ and CD8^+^ T-cells after 72 h of culture in the absence/presence of CLL-EVs as representatively shown in the flow cytometry plots on the top (gating strategy depicted with colored gates matching to the populations in the graph) and quantified in the graphs at the bottom. (**D**) Frequencies of regulatory T-cell subsets were analyzed within isolated CD4^+^ T-cells cultured for 72 h in the absence/presence of CLL-EVs based on the expression of CD25/FoxP3 (Tregs, *n* = 10) and CD49b/LAG3 (Tr1, *n* = 12) as representatively shown in the density plots and summarized in the graphs. (**E**) Surface levels of CTLA-4 and intracellular levels of TGFβ were flow cytometrically measured in isolated, activatedCD4^+^ T-cells cultured in the absence/presence of CLL-EVs for 72 h (*n* = 8). * *p* < 0.05, ** *p* < 0.01, *** *p* < 0.001, **** *p* < 0.0001. MdFI: median fluorescence intensity.

As shown in Figure 4A, expression of two T-cell exhaustion markers, i.e., PD-1 and LAG-3, was significantly increased on CD4^+^ and CD8^+^ cells in the presence of CLL-EVs. Moreover, not only was the expression of each of the exhaustion markers enhanced but also the simultaneous occurrence on the same cell was significantly more abundant as shown by the higher frequency of double positive PD1^+^/LAG3^+^ T-cells. At the same time, we also noticed an increase of KLRG-1, which is indicative for T-cell senescence (Figure 4B). Moreover, CD69, that has been associated with T-cell exhaustion in CLL [29,30], was similarly upregulated upon exposure to CLL-EVs (Figure 4C).

To analyze the frequencies of Treg subsets, we isolated CD4^+^ T-cells from HD PBMCs, cultured them for 72 h in the absence/presence of CLL-EVs, and identified conventional Tregs (CD4^+^ CD25^+^ FoxP3^+^ [31]) and Tr1 cells (CD49b^+^ LAG-3^+^ [32]) by flow cytometry. In fact, we observed a significant induction of both Treg subtypes in the presence of CLL-EVs (Figure 4D). Notably, this Treg induction was already observable under non-stimulatory conditions but more pronounced when T-cells were stimulated. Furthermore, CTLA-4 and TGFβ, two important mediators of Treg suppressiveness [31] that have previously been reported to be increased in CLL Tregs as compared to their HD-derived counterpart [29], were also significantly upregulated in the presence of CLL-EVs (Figure 4E).

These datasets show that CLL-EVs can induce both T-cell exhaustion as well as immunosuppressive Tregs potentially challenging anti-tumor immunity.

### 3.5. CLL-EVs Impair (CAR-)T-Cell Function and Express Multiple Immune Checkpoints

**Figure 5:**
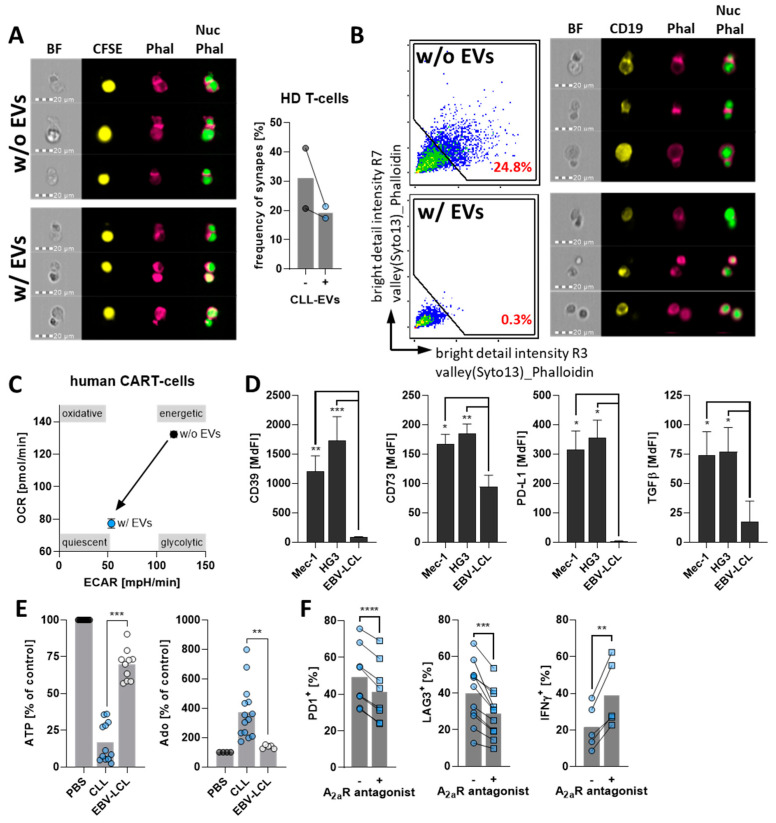

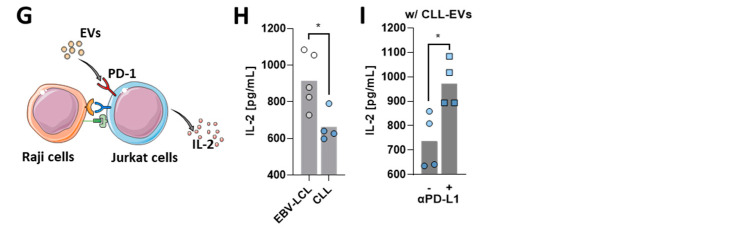
CLL-EVs impair T-cell function and express several bioactive immune checkpoints. (**A**) Immunological synapses between HD-derived T-cells and CFSE-labeled Raji cells (yellow) were analyzed via imaging cytometry after incubation in the absence (w/o EVs) or presence (w/ EVs) of CLL-EVs by the analysis of f-actin accumulation (pink, phalloidin) between nuclei (green, Nuc) and the frequency of formed synapses was quantified. (**B**) Likewise, immune synapses between CAR-T-cells and CD19-stained Mec-1 cells (yellow) were analyzed via imaging cytometry after incubation in the absence (w/o EVs) or presence (w/ EVs) of CLL-EVs by the analysis of f-actin accumulation (pink, phalloidin) between nuclei (green, Nuc). (**C**) Bioenergetics of CAR-T-cells were profiled by extracellular flux analysis using the extracellular acidification rate (ECAR, as surrogate for aerobic glycolysis) and oxygen consumption rate (OCR, as surrogate for oxphos). (**D**) Abundance of immune checkpoints was analyzed by flow cytometry on latex-bead-coupled EVs isolated from CLL cell lines (Mec-1, HG3; *n* = 7–10) and EBV-LCL lines (*n* = 5–10). (**E**) ATP consumption and adenosine production by EVs from CLL cell lines (that harbor CD39 and CD73, *n* = 12 for ATP, *n* = 14 for adenosine) and EBV-LCL lines (that have no CD39 and only low levels of CD73, *n* = 10 for ATP, *n* = 5 for adenosine) were analyzed using an enzymatic assay and compared in relation to a control without EVs (PBS, set as 100%). (**F**) Frequencies of T-cells positive for the exhaustion markers PD1 (*n* = 8) and LAG3 (*n* = 11) as well as the cytokine IFNγ (*n* = 5) were analyze by flow cytometry of activated, CLL-EV-treated T-cells cultured for 72 h in the absence/presence of the A2a receptor antagonist ZM241385. (**G**) Schematic picture of the Raji/Jurkat co-culture model system to analyze the effect of PD-L1 on the CLL-EVs. (**H**) Supernatants of Raji/Jurkat co-cultures treated with EVs isolated from EBV-LCL lines (that do not carry PD-L1, *n* = 5) or CLL cell lines (that have high levels of PD-L1, *n* = 4) were analyzed for IL-2 by means of sandwich ELISA. (**I**) Supernatants of Raji/Jurkat co-cultures treated with CLL-EVs in the absence/presence of a PD-L1-blocking antibody were analyzed for IL-2 by means of sandwich ELISA (*n* = 4). Error bars show the standard error mean. * *p* < 0.05, ** *p* < 0.01, *** *p* < 0.001, **** *p* < 0.0001. MdFI: median fluorescence intensity, BF: bright field, Phal: phalloidin, Nuc: nucleus.

Next, we sought out to explore the potential impact of CLL-EVs on T-cell function. In fact, several T-cell dysfunctions have been described in CLL, including the inability to form immunological synapses [33,34]. Mechanistically, immune synapse assembly requires remodeling of the cytoskeleton with accumulation of F-actin at the site of cell–cell contact [35] that can be detected using phalloidin. Imaging cytometry together with a machine-learning algorithm enabled us to quantify the frequency of formed immune synapses between T-cells and Raji target cells (pre-activated with Staphylococcal enterotoxin A) based on phalloidin accumulation at the intercellular junctions. We found that properly formed synapses were vastly reduced upon treatment with CLL-EVs (Figure 5A).

CAR-T-cells represent a promising therapeutic option for CLL. However, several issues including early exhaustion after transfusion remain to be fully addressed [36]. Therefore, we also tested the CLL EVs’ impact on anti-CD19 CAR-T-cells with 4-1BB costimulatory domain that were generated using T-cells isolated from a CLL patient. First, we confirmed that CLL-EVs are taken up by or attach to CD19 CAR-T-cells (Supplementary Figure S3A). Next, we evaluated synapse formation and observed a complete abrogation in response to CLL-EV treatment (Figure 5B), which could very well explain why CLL-EVs reduce the CAR-T-cells’ killing efficacy as recently reported [17]. Furthermore, we could detect induction of exhaustion (Supplementary Figure S3B) and metabolic quiescence (Figure 5C, Supplementary Figure S3C), both of which are hallmarks of CAR-T-cell failure in CLL [36].

During our analyses to identify the underlying mechanisms, we found that CLL-EVs harbor a multifaceted palette of immunological checkpoints (ICs) including CD39, CD73, PD-L1, TGFβ, Gal9, and CD112 (Figure 5D, Supplementary Figure S4A,B). All detected ICs were significantly more abundant on CLL-EVs compared to EVs isolated from EBV-LCL lines. CD39 and CD73 are two ectonucleotidases that convert ATP/ADP to AMP, and AMP to adenosine, respectively [37]. The latter has been already intensively described to be suppressive via the A2a receptor on the T-cell surface [38,39]. In fact, by incubating isolated CLL- and EBV-LCL-EVs with ATP we found that the EVs from CLL lines consume significantly greater amounts of ATP and convert it to significantly greater amounts of adenosine (Figure 5E). This ATP consumption was blocked by an inhibitor of CD39 (Pom1), while adenosine production was blocked by CD73 inhibition (APCP) (Supplementary Figure S4C). These results indicate that those two ICs on CLL-EVs are enzymatically active. In line with this, T-cells cultured in the presence of CLL-EVs showed an increased phosphorylation of CREB (Supplementary Figure S4D), which is a downstream target of the A2a receptor signaling [38]. Moreover, exhaustion of CLL-EV-treated T-cells as well as cytokine production was at least partly rescued by use of an A2a receptor antagonist (Figure 5F). The role of PD-L1-mediated immune suppression is well documented in CLL [40,41] and we wanted to determine bioactivity of PD-L1 on CLL-EVs. To this end, we used a model system of Raji cells (targets) and Jurkat T-cells (effectors) based on IL-2 secretion (Figure 5G). Indeed, IL-2 secretion by Jurkat T-cells was significantly reduced in the presence of CLL-EVs as compared to EBV-LCL-EVs (Figure 5H). Ultimately, IL-2 secretion was rescued when interfering with the PD-1–PD-L1 interaction using a PD-L1-blocking antibody in the presence of CLL-EVs (Figure 5I).

Taken together, here we demonstrated that CLL-EVs functionally impact (CAR-)T-cells and that a number of ICs found on CLL-EVs could be responsible for this. Thus, targeting ICs individually in CLL might not be the appropriate strategy. This needs to be further investigated.

## 4. Discussion

T-cells play a key role for CLL immune surveillance and CLL pathogenesis as well as in preventing infectious complications during disease progression and increasing immune deficiency [34]. Numerous defects and dysregulations have been described, including improper formation of immune synapses [33,42], impaired responses to stimulatory mitogens [43,44], signs of chronic activation [45,46] with increased exhaustion [47,48], and a generally insufficient mounting of an anti-tumor immune response [49]. It is also evident that many of those T-cell dysfunctions are provoked by recurrent cell-to-cell contact with CLL-cells [29,50], which often involve ICs such as PD-L1 [40,41]. However, little is known about cell-to-cell-contact-independent mechanisms, especially in terms of EVs, although CLL-EVs have been shown to have prognostic potential [51]. Moreover, tumor-derived EVs have already been shown to reprogram monocytes [15,52] and stroma cells [53] in the context of CLL. Especially in light of the emerging relevance of applying immunotherapies such as CAR-T-cells in CLL [36], it is of utmost importance to further improve our understanding of the immunomodulatory role of CLL-EVs. Therefore, we performed a comprehensive analysis of effects mediated by CLL-EVs on T-cells, their functional consequences, and the potential involvement of CLL-EV-carried ICs.

First, we confirmed the capability of T-cells to bind and to readily take up CLL-EVs, as already indicated in earlier studies [12]. Although previous experiments by others conclude the importance of CLL-EV uptake [12], our data suggest that not only uptake but also surface interaction (possibly via ICs receptors, see below) is occurring. Unfortunately, we were not able to delineate the balance between surface ligation and uptake of CLL-EVs on the observed T-cell dysfunctions because available inhibitors for EV uptake are cytotoxic to T-cells over longer in vitro culture periods. Nevertheless, we were still able to show that a substantial amount of CLL-EVs was engulfed via endocytosis because 1) the frequency of EV^+^ T-cells was reduced when including endocytotic inhibitors, and 2) the majority of T-cells incubated with fluorescently labeled CLL-EVs still retained a strong fluorescent signal even when surface-bound EVs were removed with trypsin prior to the analysis. Notably, EV attachment/uptake was most prominent when using activated T-cells, which might be caused by a general upregulation of surface receptors upon TCR stimulation and requires further investigation.

The dual role of T-cells for CLL pathogenesis and development has been intensively reviewed recently [29,34]. T-cells in the tumor microenvironment seem to provide nurturing and/or survival signals to the CLL-cells, and their ability to mount an anti-tumor response is limited. However, underlying mechanisms remain largely inconclusive. Our analyses reveal the ability of CLL-EVs to interfere with T-cell function on several levels similar to effects observed by EVs from other models/tumor entities (as recently reviewed [54]): (1) the viability and proliferation of T-cells are drastically reduced, (2) T-cell activation and effector cytokine secretion are diminished, (3) T-cell metabolism is shifted towards a quiescent state with an according shift of the effector/memory subsets, and (4) T-cell exhaustion is fostered. Another hallmark in T-cell dysregulation of CLL is the accumulation of Treg subsets that correlate with poor prognosis of CLL patients [55,56]. Again, CLL-EVs may be a driving factor as we showed an increase in regulatory T-cell subsets together with the increased expression of inhibitory mediators upon presence of CLL-EVs. Whether the increased frequencies are a result of Treg induction or rely more on the preferential cytotoxic effect on conventional T-cells remains speculative. However, CD4^+^ and CD8^+^ T-cells are similarly affected in terms of reduced viability by CLL-EVs, and CLL-EVs carry large amounts of TGFβ, which is known to induce Tregs [57]. Both findings favor the hypothesis of an active induction of Tregs by CLL-EVs, which certainly needs intensified investigation. Ultimately, we identified CLL-EVs as one factor causing improper formation of immune synapses, which is a known contributor to CLL immune escape [33].

Impaired immune surveillance, as observed in CLL, can be attributed to the aberrant expression of IC receptors on CLL T-cells and the abundant expression of the corresponding ligands on the CLL-cells [7]. In accordance with previous findings of the increased expression of IC on CLL-cells, we found multiples of those ICs being carried by the CLL- EVs, which could explain the observed T-cell dysfunctionalities. Interestingly, ICs were previously identified as a relevant promoter of immune synapse malformation [42]. In the latter study by Ramsay and colleagues and in contrast to our data, cell-to-cell contact between CLL-cells and T-cells was a prerequisite for dysfunctional immune synapses. However, since we measured large amounts of ICs on CLL-EVs it appears likely to us that CLL-EVs can mimic the parental CLL-cell surface in this regard resulting in comparable synapse anomalies. Additionally, the ectonucleotidases CD39 and CD73, which convert ATP/ADP to AMP and further to adenosine, are both highly expressed by CLL-cells, especially in the proliferating compartment [37]. Similarly, we found a high abundance of those two checkpoints on the CLL-EVs and could confirm their enzymatic activity. This is of particular interest as adenosine in CLL has a dual-role: (1) it supports and protects the CLL-cells [58] and (2) it impairs T-cell function [59]. In the latter case, different modes of action were found, including reduced cytokine expression [60], impaired T-cell metabolism [38], induction of Tregs [61], and induction of T-cell exhaustion [62]. Thus, it is not surprising that all the phenomena that were previously observed for adenosine on T-cells were also evident in T-cells that we treated with CLL-EVs. Additionally, the role of PD-L1 as an IC in the context of T-cell inhibition and anti-tumor therapy is indisputable [63,64]. PD-L1 expressed by CLL-cells can inhibit T-cell activation [41], suppress the secretion of effector cytokines by T-cells [65], enhance T-cell exhaustion, and impair the formation of immune synapses [40]. Again, those effects could also be observed in the presence of PD-L1-harboring CLL-EVs. However, to date, single anti-PD1/PD-L1 treatment in CLL therapy has an unsuccessful history [66,67]. So far, the reason for the unresponsiveness towards a blockade of the PD-1/PD-L1 axis in CLL, albeit promising results from preclinical models [40,65], is vastly unknown. In fact, our data, which of course requires experimental verification, could contribute to speculation regarding the possible causes: the great variance of ICs such as the aforementioned CD39/CD73 and TGFβ but also galectin-9 (a ligand for TIM-3 and PD-1 [68]) and CD112 (a ligand for TIGIT [69]) may compensate for the blockade/inhibition of one single IC (such as PD-L1). Many downstream effects of those co-inhibitory signals are shared and their concerted action may act in an additive or even synergistic manner. This notion is corroborated by studies demonstrating that the dual blockade of PD-1 and LAG3 ICs could limit CLL development and restore immune competence while an individual IC-targeting was inefficient [70]. Moreover, the high abundance and constant secretion of EVs (that harbor PD-L1) in CLL may simply outcompete the blocking activity of anti-PD-1/PD-L1 antibodies. In fact, acquired resistance to anti-PD-1/PD-L1 therapy by PD-L1-carrying EVs has already been demonstrated in several solid-tumor entities [71] and it is speculated that PD-L1 presented by EVs may preferentially bind immunotherapeutic antibodies and thereby act as a “sink” to divert them away from the tumor cells [72]. This argument is strengthened by the fact that the Bruton tyrosine kinase inhibitor ibrutinib is able to reduce secretion of CLL-EVs [11] and at the same time to increase efficacy of PD-1/PD-L1 blockade [73,74]. Currently a combination of ibrutinib and PD-1 blockade is being evaluated within a phase II clinical trial in CLL patients [75].

Importantly, although CD19-targeting CAR-T-cell therapies in CLL show promising response rates [76], long-term results remain limited [77,78]. Key mechanisms responsible for insufficient CAR-T-cell efficacy include loss of the target antigen [79], T-cell defects [80], and an immunosuppressive tumor microenvironment [81]. In addition, CAR-T-cells produced from CLL patients show increased signs of exhaustion compared to those derived from healthy donors before infusion [82]. In particular, those patients whose CAR-T-cells expressed multiple inhibitory receptors such as PD-1, TIM-3, and LAG-3 presented lower responses [78]. A recent study identified EVs from CLL patient plasma to be capable of impairing antigen-specific proliferation and killing, and of inducing CAR-T-cell exhaustion [17], which we could confirm as well. Moreover, our analyses append the CLL-EV-mediated repertoire of CAR-T-cell dysfunction by the almost absent formation of immune synapses and the drastically reduced cellular metabolism. To sum up, CAR-T-cell therapy in CLL could be affected by CLL-EVs in two ways: (1) the CLL T-cells’ lack of functional competence has a substantial impact on the final CAR-T-cell product [82], and (2) re-infused CAR-T-cells again have to face immunosuppressive CLL-EVs.

## 5. Conclusions

In conclusion, we showed that CLL-EVs can be a mediator of T-cell dysfunction in CLL. In fact, CLL-EVs carry multiple ICs as a potential further tool for promoting an immune-privileged environment mediated by CLL. Moreover, various studies indicate that targeting individual ICs in CLL might not be sufficient to reach meaningful clinical effects. The numerous ICs on CLL-EVs could be one of the underlying causes (over-)compensating the blockade of a single IC by their coordinated and combined activity. Therefore, our findings could pave the way for novel therapeutic strategies in CLL, such as the simultaneous blockade of multiple ICs or the targeting of EV production and secretion by CLL-cells. A better understanding of immune-suppressive strategies could help to foster intrinsic anti-tumor immunity and increase the efficacy of adoptive cell therapies, most importantly CAR-T-cells.

## Data Availability

Original data will be made available by the corresponding author upon reasonable request.

## Supplementary Materials

The following supporting information can be downloaded at: https://www.mdpi.com/article/10.3390/cells11142176/s1. Figure S1: Supportive data for Figure 1. Figure S2: Bioenergetics of CLL-EV-treated T-cells. Figure S3: Effects of CLL-EVs on CAR-T-cells. Figure S4: Immune checkpoint activity on CLL-EVs. Table S1: Experimental inhibitors. Table S2: Antibodies for flow cytometry and fluorescence microscopy.
