# Supplementary material for: CLL-Derived Extracellular Vesicles Impair T-Cell Activation and Foster T-Cell Exhaustion via Multiple Immunological Checkpoints
Source: Cells. 2022 Jul 12;11(14):2176. doi: 10.3390/cells11142176 (PMC9320608; doi:10.3390/cells11142176)
Supplement: Supplementary file 1 [file cells-11-02176-s001.zip › cells-1774547_Suppl Figures.pdf]

Suppl. Figure S1

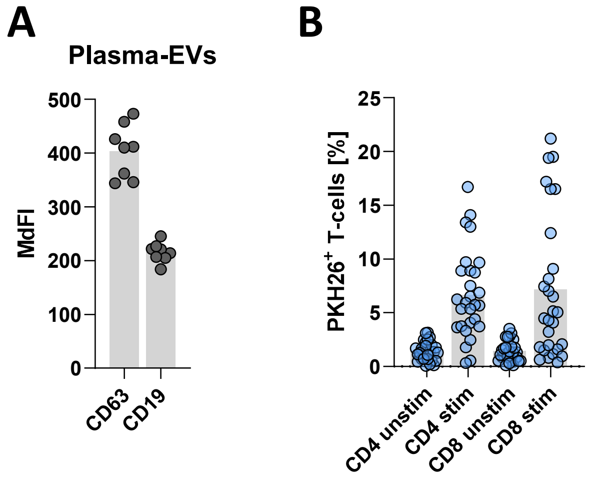

Suppl. Figure S1: (A) EVs isolated from CLL patient plasma were analyzed for the EC marker CD63 and the parental cell marker CD19 by flow cytometry. **(B)** Uptake of PKH26-labeled CLL-EVs in CD4$^+$ and CD8$^+$ T-cell subsets was analyzed after 24-72h of culture by flow cytometry. **(C)** Representative picture of fluorescent microscopy of activated CD4$^+$ and CD8$^+$ T-cells (green) cultured for 72h in presence of PKH26-labeled CLL-Evs (red) with a nuclear counterstain using DAPI (blue).

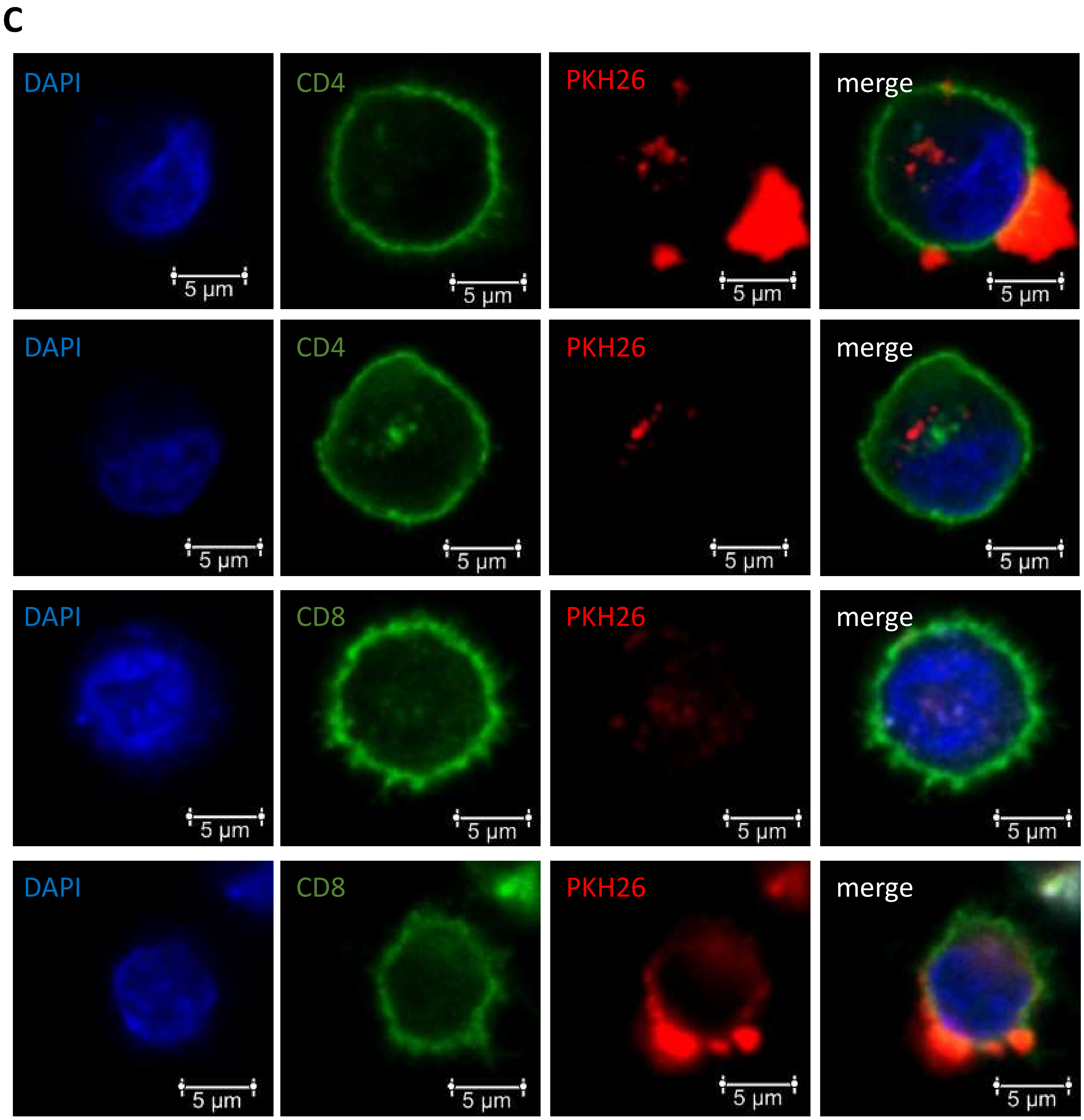

Suppl. Figure S2

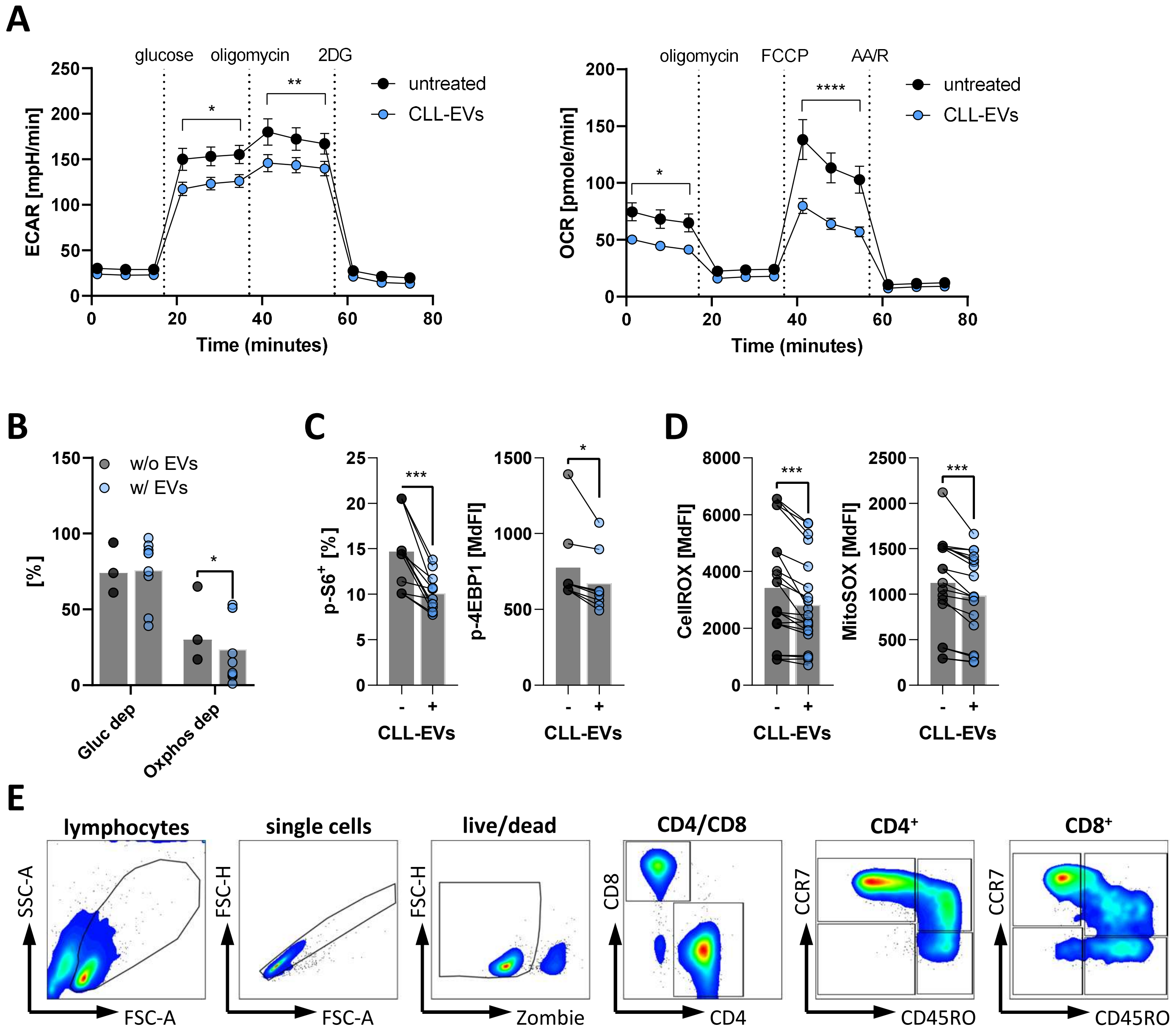

Suppl. Figure S2: (A) Bioenergetic kinetics of HD-derived, activated T-cells cultured in absence/ presence of CLL-EVs for 72h were measured by a glycolysis stress test (left, n=3) and a mitochondrial stress test (right, n=3) using sequential injection of metabolically active inhibitors as described in materials and methods. **(B)** Quantification of glucose and oxphos dependency of activated T-cells cultured in absence/presence of CLL-EVs for 72h was measured by Scenith (n=9). **(C)** mTOR downstream signaling (phospho-S6, n=12; phospho-4EBP1, n=8) was analyzed by PhosFlow in activated T-cells cultured for 24h in absence/presence of CLL-EVs. **(D)** Cellular ROS levels (CellROX, n=20) and mitochondrial superoxides (MitoSOX, n=18) were analyzed by flow cytometry in activated T-cells cultured for 72h in absence/presence of CLL-EVs. **(E)** Gating strategy of naïve, central memory, effector memory, and terminally differentiated effector T-cells within the CD4+ and CD8+ compartment. Error bars show the standard error mean. * $p<0.05$, ** $p<0.01$, *** $p<0.001$, **** $p<0.0001$. MdFI: median fluorescence intensity.

Suppl. Figure S3 – human CART-cell product

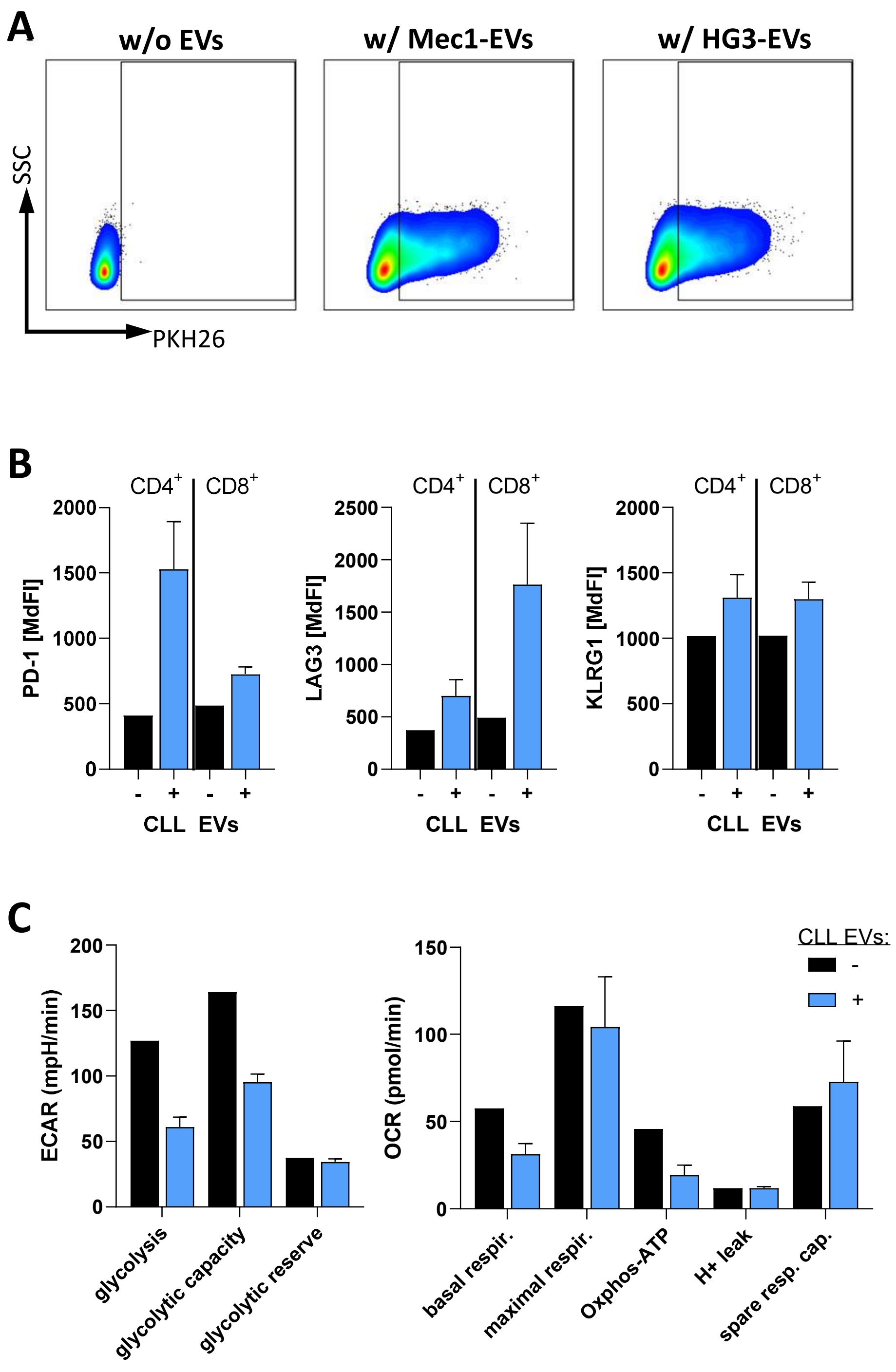

Suppl. Figure S3: **(A)** Representative dot plots of CART-cells incubated in absence (w/o EVs) or presence (w/ EVs) of PKH26-labeled CLL-EVs for 24h. **(B)** Surface expression of exhaustion markers was measured on CD4^{+} and CD8^{+} CART-cells cultured in absence/ presence of CLL-EVs for 72h. **(C)** Bioenergetics of CART-cells cultured for 72h in absence/ presence of CLL-EVs was analyzed on a Seahorse XFe96 and calculated using the Wave software. Error bars show the standard error mean. MdFI: median fluorescence intensity.

Suppl. Figure S4 – Immune checkpoints on CLL-EVs

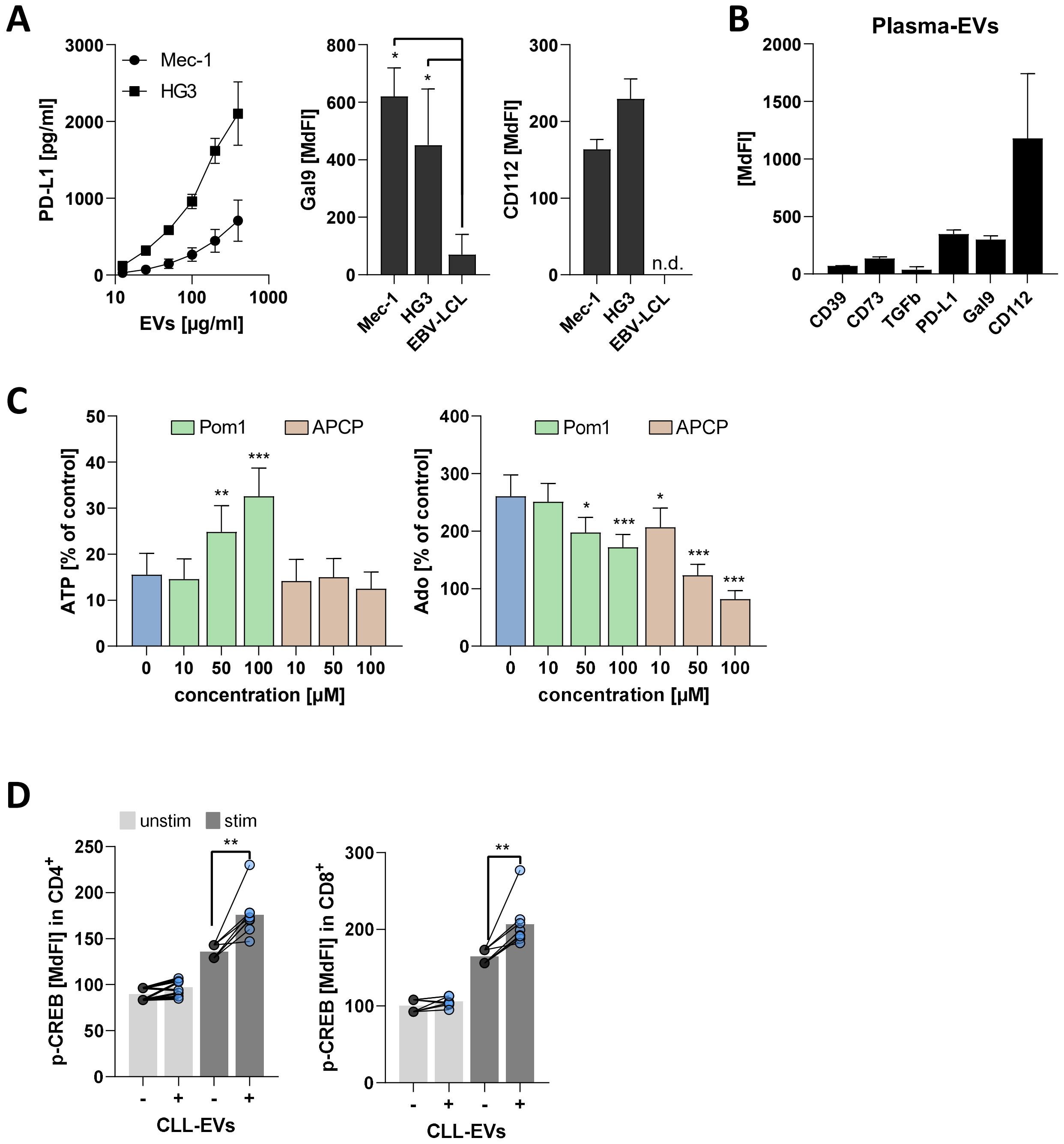

Suppl. Figure S4: (A) PD-L1 levels were determined on increasing concentrations of CLL-EVs using a commercial PD-L1 ELISA (Human PD-L1 [28-8] SimpleStep ELISA®Kit, abcam) according to the manufacturer's recommendations (n=3). The immune checkpoints Gal9 (n=4) and CD112 (n=5) were analyzed on CLL-EVs compared to EBV-LCL-EVs by flow cytometry using latex beads as described in materials and methods. **(B)** Immune checkpoints were analyzed on EVs isolated from CLL patient plasma by flow cytometry using latex beads as described in materials and methods (n=3). **(C)** ATP consumption and adenosine production by EVs from CLL celll lines were analyzed using an enzymatic assay and compared in relation to a control without EVs (PBS, set as 100%) upon increasing concentrations of the CD39 inhibitor POM1 and the CD73 inhibitor APCP (n=8). **(D)** CREB signaling was analyzed by PhosFlow in activated $CD4^+$ and $CD8^+$ T-cells cultured for 24h in absence/presence of CLL-EVs (n=8). Error bars show the standard error mean. * $p<0.05$, ** $p<0.01$, *** $p<0.001$, n.d. not detectable. MdFI: median fluorescence intensity.
