# Supplementary material for: CLL-Derived Extracellular Vesicles Impair T-Cell Activation and Foster T-Cell Exhaustion via Multiple Immunological Checkpoints
Source: Cells. 2022 Jul 12;11(14):2176. doi: 10.3390/cells11142176 (PMC9320608; doi:10.3390/cells11142176)
Supplement: Supplementary file 1 [file cells-11-02176-s001.zip › cells-1774547_Supplemental Tables_revised.pdf]

Supplemental Table S1: Inhibitors

Compound	Manufacturer	Order No.	Concentration
**APCP**	Tocris	3633	10-100 µM
**Cytochalasin D**	Cayman Chemicals	11330	5 µM
**Dynasore**	Cayman Chemicals	14062	20 µM
**EIPA**	Cayman Chemicals	14406	20 µM
**GW4869**	Selleckchem	S7609	10 µM
**Heparin**	Sigma-Adrich	H3149	10 µg/mL
**Nexinhib20**	Tocris	6089	6 µM
**POM 1**	Tocris	2689	10-100 µM
**ZM241385**	Selleckchem	S8105	30 µM

Supplemental Table S2: Fluorochrome-conjugated antibodies

Antibody	Clone	Manufacturer
**CD4**	OKT4	Biolegend
**CD8**	RPA-T8	Biolegend
**CD25**	M-A251	BD Biosciences
**CD25**	BC96	Biolegend
**CD36**	REA760	Miltenyi biotec
**CD39**	A1	Biolegend
**CD45RO**	UCHL1	Biolegend
**CD49b**	eBioY418	ThermoFischer Scientific
**CD57**	HCD57	Biolegend
**CD62L**	DREG-56	Biolegend
**CD73**	AD2	Biolegend
**CD112**	TX31	Biolegend
**CD137**	4B4-1	Biolegend
**CD152 (CTLA-4)**	L3D10	Biolegend
**CD154 (CD40L)**	24-31	Biolegend
**CD223 (LAG3)**	11C3C65	Biolegend
**CD274 (PD-L1)**	29E.2A3	Biolegend
**CD279 (PD-1)**	EH12.2H7	Biolegend
**FoxP3**	206D	Biolegend
**Galectin-9**	9M1-3	Biolegend
**GLUT1**	202915	R&D Systems
**GLUT3**	202017	R&D Systems
**Granzyme B**	GB11	Biolegend
**IFNγ**	4S.B3	Biolegend
**Ki67**	Ki67	Biolegend
**KLRG1**	14C2A07	Biolegend
**p-4EBP1**	M31-16	BD Bioscience
**p-AKT**	M89-61	BD Bioscience
**p-AMPK**	polyclonal	Biorbyt
**p-CREB**	J151-21	BDBioscience
**p-mTOR**	O21-404	BD Bioscience
**p-S6**	N7-548	BD Bioscience
**Puromycin**	12D10	Sigma Aldrich
**TGFβ**	CH6-17E5.1	Miltenyi biotec

All conjugated antibodies were used at the manufacturers' recommended dilutions.
